# P03-028 – Spectacular efficiency of isotretinoin in small doses in the sebopsoriasis treatment

**DOI:** 10.1186/1546-0096-11-S1-A226

**Published:** 2013-11-08

**Authors:** M Mael-Ainin, H Boudhir, A Saidi, K Senouci, B Hassam

**Affiliations:** 1 Dermatologie - Vénérologie, Centre Hospitalier Universitaire IBN Sina, Morocco; 2 Centre of Anatomic Pathology, United Nations, Rabat, Morocco

## Introduction

Sebopsoriasis is a chronic erythematous scaly dermatitis that is inconveniencing due to its showing character and the frequent inefficiency of the various treatments used. We report through this case the efficiency of low-dose isotretinoin in this particular form of psoriasis.

## Objectives

To emphasize on isotretinoin low-dose efficiency in sebopsoriasis treatment.

## Methods

Miss H.B, 30 years old, with a history of a psoriasis in the father. The patient was followed up for two years for a histologically confirmed sebopsoriasis. She received various therapies, initially put on moisturizers and Imidazoles but with no improvement, then on dermocorticoids and vitamin D derivatives. Treatment with UVB phototherapy could not be achieved, given the professional constraints of the patient. She was put on Methotrexate at 12.5 mg/week dose for six months without improvement. Treatment based on 5mg Isotretinoin, three times per week, was recommended allowing the complete whitening of lesions two months after the start of treatment with the benefit of one year hindsight.

## Results

The sebopsoriasis alters significantly the quality of life of patients. The treatment is mainly based on local treatments but those are often ineffective. Phototherapy or other systemic therapies are also used with varying results. These centro-facial effects during psoriasis respond well to small doses of isotretinoin at the rate of 5 mg three times per week or every day, which is well illustrated through this observation.

## Conclusion

Our case shows a remarkable efficiency with a good tolerance of isotretinoin in sebopsoriasis treatment. This promising result should prompt us to use more this retinoid in the treatment of this particular form of psoriasis.

## Disclosure of interest

None declared

